# College of Surgeons

**Published:** 1830-04-01

**Authors:** 


HUNTERIAN ORATION. By Mr. GUTHRIE.

This was the first and the only ORATION which we, or perhaps any of our readers, have ever heard within the walls of the College of Surgeons. A written disquisition has usually been designated by the title of oration—and we do not find fault with such a procedure. We have but few orators among us, and there is probably not another surgeon on the rolls of the College who could have delivered an oration without other notes or memoranda than the flight of time, as indicated by the hand of the silent monitor on the table. The discourse occupied an hour—was delivered in language that might have been, and, indeed, has been, committed to the press without the necessity of a single correction—and was spoken with as much ease and fluency, as Mrs. Siddons was wont to read passages from the best English authors. Mr. Guthrie's talent in this way is inimitable, and called forth, without the possibility of repression, repeated bursts of applause. After eulogizing the talents, the genius, and, above all, the INDUSTRY, of John Hunter, the orator recapitulated, in a summary but eloquent manner, the various improvements in operative and medical surgery which have taken place since the death of the illustrious founder of the museum. This procedure, he conceived, would prove the most grateful incense which could be offered at the shrine of the illustrious dead—if disembodied spirits were permitted to witness the scenes that occurred on the stage they had quitted. Living and contemporary authors were allotted their just meed of praise—and the names of Abernethy, Cooper, Brodie, Bell, White, Copeland, and Earle, most of whom were present, were honorably and liberally mentioned, in connexion with the improvements which they had introduced into the practice of surgery.

It is asserted by the lying Lancet, that at the mention of Mr. Earle's name, there were marks of disapprobation among the audience! We appeal to each and every individual present, as to the mendacity of this assertion. It is only equalled by the astounding ignorance of Churchwarden Wakley, who has made RUYSCH a famous Dutch LITHOTOMIST!! The fellow knows no more of RAU than he knows of the incarnations of Vishnu.

